# Clinical presentations, diagnosis, management, and outcomes of renal mucormycosis: An overview of case reports

**DOI:** 10.3389/fmed.2022.983612

**Published:** 2022-08-24

**Authors:** Mojtaba Didehdar, Zahra Chegini, Amin Khoshbayan, Alireza Moradabadi, Aref Shariati

**Affiliations:** ^1^Department of Medical Parasitology and Mycology, Arak University of Medical Sciences, Arak, Iran; ^2^Department of Microbiology, School of Medicine, Hamadan University of Medical Sciences, Hamadan, Iran; ^3^Department of Microbiology, School of Medicine, Iran University of Medical Sciences, Tehran, Iran; ^4^Molecular and Medicine Research Center, Khomein University of Medical Sciences, Khomein, Iran

**Keywords:** renal, kidneys, mucormycosis, COVID-19, invasive fungal

## Abstract

**Background:**

Renal mucormycosis (RM) is a rare presentation of invasive mucormycosis with a high mortality rate. There is no single systematic review of the literature that indicates the different clinical aspects of RM.

**Methods:**

A systematic search of PubMed/Medline was performed to collect individual case reports of RM in patients of all ages published between 2010 and April 2022.

**Results:**

Seventy-one individual cases were detected through PubMed bibliographic database searches, with a final assessment performed on 60 patients with RM. India and Asia had the largest number of reported cases, with 30 (50%) and 42 (70%) reports, respectively. Also, 74 and 26% of the patients with a mean age of 33 years were male and female, respectively. RM showed 44% mortality rate in the analyzed cases. Immunosuppressive agent therapy followed by tissue transplantation (kidney and liver) and diabetes were the most remarkable risk factors in patients. Nevertheless, 22% of the patients were immunocompetent with no apparent underlying condition. COVID-19 positivity was detected in eight adult patients with an 87% mortality rate. The most common signs of infection were fever, flank pain, and oliguria; additionally, isolated RM was reported in 57% of the cases. In 55% of the patients, histopathologic examination alone was sufficient to diagnose RM, whereas molecular methods and culture were used in only 18 and 35% of patients, respectively. Surgery alone, surgery plus anti-infection therapy, and anti-infection therapy alone were used in 12, 60, and 13% of patients, respectively. Furthermore, 15% of the patients died before any treatment.

**Conclusion:**

The early diagnosis of RM is necessary. In this regard, the use of molecular-based diagnostic assays can help identify the fungus at the genus and species levels and use an appropriate treatment in the shortest possible amount of time. Because of the increase in antibiotic resistance in recent years, determining microbial susceptibility tests can lead to the better infection management. Additionally, withdrawal of immunosuppressant, appropriate surgical intervention, and antifungal therapy are the main factors associated with a successful outcome in RM.

## Introduction

Mucormycosis, previously called Zygomycosis, comprises a wide range of invasive infections caused by different *Mucorales* species such as *Rhizopus, Apophysomyces, Lichtheimia Mucor, Rhizomucor, Cunninghamella*, and *Saksenaea* (1, 2). Mucormycosis has been considered as a rare fungal infection but has a high mortality rate (3, 4). Patients with underlying conditions such as tissue transplantation, diabetes, and immunosuppressive agent's therapy are more at risk of developing mucormycosis (5). The common mucormycosis clinical presentations are rhino-orbito-cerebral (ROCM), pulmonary, cutaneous, gastrointestinal (GI), and disseminated forms.

Kidney involvement, renal mucormycosis (RM), has been reported in up to 20–22% of the cases with disseminated forms (5, 6). The exact mechanism of RM is not clear yet; however, retrograde spread from lower urinary tract infection and blood dissemination to the kidneys have been suggested (7). Thus, in high-risk patients, early demonstration of the infection in the urine and cystoscopy assessment of the bladder could be helpful to avert dissemination as bladder can also be a portal of entry for *Mucorales* (8).

*Mucorales* have angioinvasive ability and invade the blood vessels, thus leading to vascular thrombosis and related ischemic necrosis of the kidney. Furthermore, these fungi could also invade the tubules, the glomeruli, and the parenchyma, in addition to the renal vessels. Collectively, medullary and cortical necrosis leads to renal failure and irreversible kidney damage (8, 9).

Note that the psoas and quadratus lumborum muscles posteriorly and with the peritoneal contents anteriorly provide good protection for kidneys. However, the kidneys could sustain lacerations and contusions from lower chest or blunt abdominal trauma, as occurs not infrequently in cases of high-impact motor vehicle crashes (10). Another study also reported that following intravenous inoculation in mice, the predominant sites of focal infection were the kidney and brain (11). Accordingly, different surgical interventions and traumas might have led to the entrance of the *Mucorales* into the patients' kidneys. Further, an intravascular catheter should be considered as a common portal of Mucorales' entrance to the body which could lead to the nosocomial disseminated mucormycosis (1, 12, 13).

Because of the lack of a comprehensive review of the literature indicating the different clinical perspectives of RM, this review paper was performed to compile available data about this infection and its patients in order to help in their diagnosis as well as management procedure.

## Methods

### Literature search

The Medline (*via* PubMed) was searched from January 1, 2010, to April 29, 2022, where Boolean Operators were used to extracting search keywords from the National Library of Medicine's Medical Subject Heading (MeSH) terms, abstracts, or titles (and, or): “*Mucor*” or “*Mucorales*” “*Rhizopus*” or “*Lichtheimia*” or “*Rhizomucor*” or “*Absidia*” or “*Cunninghamella*” or “*Apophysomyces*” or “Mucormycosis” or “Zygomycosis” and “Renal” or “Kidney.” Further, we also evaluated the references of the found articles and cross-checked for any other cases that may have been overlooked or missed during the initial investigation. Non-English written papers were excluded. Hickey et al. study, as well as our most recent publications, were used as the protocol for this study (14–16).

### Inclusion criteria

In the current review paper, RM individual case reports in patients of all ages published in English between 2010 and April 2022 and available online in Medline (*via* PubMed) were included. Analysis of the included studies was performed carefully by both authors (MD and AS).

### Exclusion criteria

**Figure 1:**
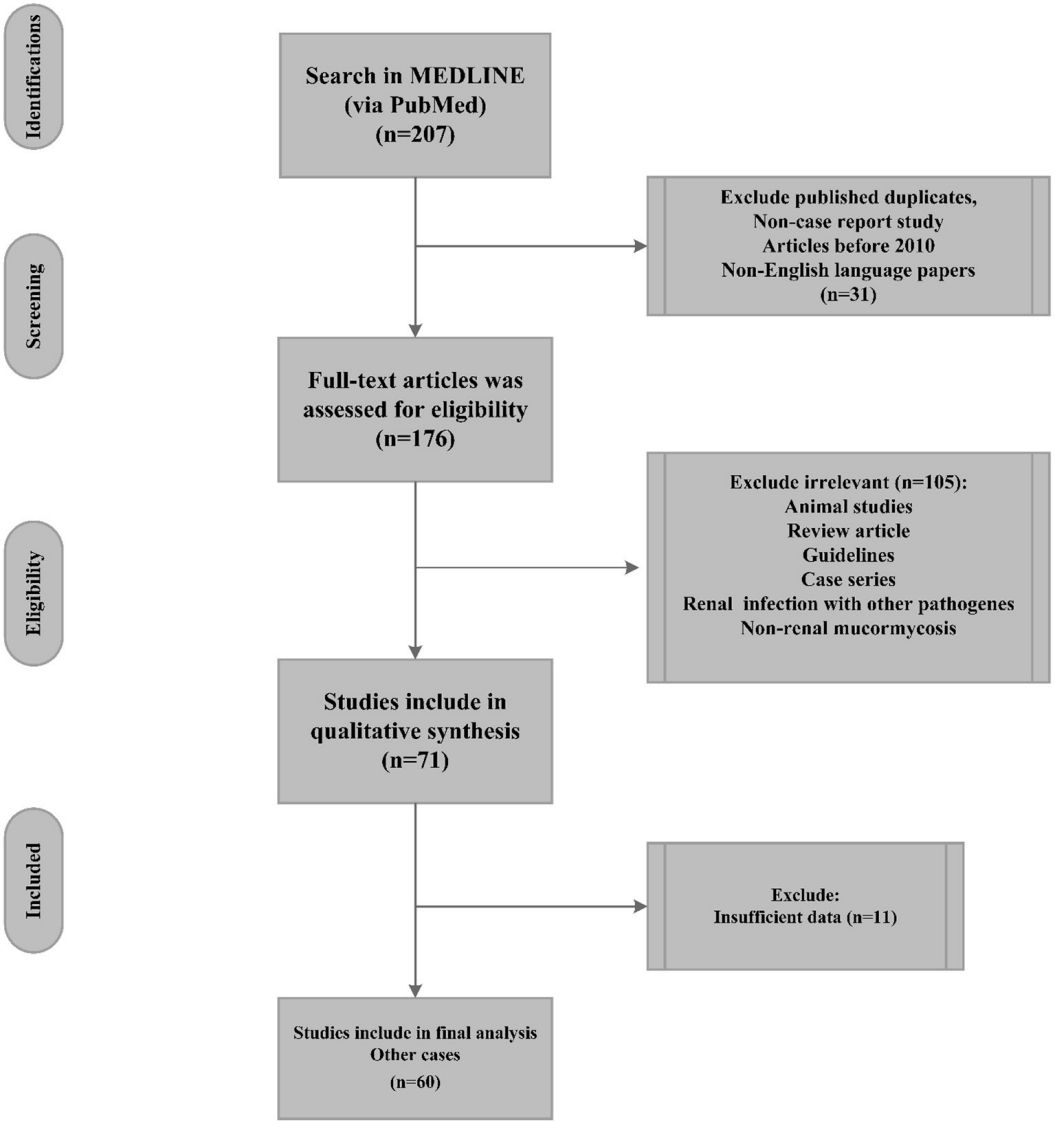
Flow chart of renal mucormycosis publication selection (PubMed reported cases 2010 until April 2022) and their inclusion in the systematic review.

Guidelines, review articles (systematic or meta-analysis), renal infections resulting from other pathogens, animal models, and studies with insufficient reported data were excluded from the final analysis (Figure 1).

### Study selection and data extraction

MD and AS evaluated the articles, and in case of a discrepancy, both authors were obligated to inspect the paper for eligibility secured for review. Individual case reports were assessed to elicit data about the epidemiological features, clinical signs, diagnostic methods, and infection management of RM in patients of all ages. In the end, Excel software (Microsoft, Redmond, WA, USA) was performed to collect and evaluate the following information extracted from articles: publication year, country, age/gender, underlying conditions, causative *Mucorales*, clinical signs, surgical intervention, antifungal treatment, and outcomes.

### Quality assessment

The Joanna Briggs Institute's (JBI) critical appraisal checklist was used for the quality assessment of studies (17).

## Results

### Epidemiology

Seventy-one cases, as individual case reports, were detected *via* searches of the PubMed bibliographic database; afterward, 11 cases were eliminated from the final analysis because of insufficient data or non-RM (Figure 1). Accordingly, sixty cases were included in the present study and were used for final analysis. The geographical distribution of the detected cases was as follows: India (30 cases), USA (nine cases), China (four cases), and Japan (three cases). Other countries, including Saudi Arabia, France, Germany, Korea, Netherland, Qatar, Romania, and South Africa, reported only one case. Thus, Asia had the largest number of cases (70%, 42 cases), followed by the Americas (15%, 9 cases) and Europe (10%, six reports). In this regard, only two and one reports were detected from Australia and Africa, respectively. In addition, 74 and 26% of the patients with a mean age of 33 years (range: 0.25–76, SD: 19.22 years) were male and female, respectively. The analyzed cases had a 44% mortality rate.

According to our analysis, 11 patients (11/60, 18%) had blood disorders, including acute myelogenous leukemia (AML), acute lymphocytic leukemia (ALL) (five cases), non-Hodgkin's lymphoma and Idiopathic CD4 lymphocytopenia, as well as anemia (three cases). Notably, one of these patients had a history of Hematopoietic Stem Cell Transplantation (HSCT) and two deaths occurred due to RM. Additionally, immunosuppressive agent therapy, tissue transplantation (kidney and liver), diabetes, and chemotherapy were reported in 27, 20, 18, and 14% of the patients, respectively.

In addition to the mentioned underlying conditions, different disorders such as Behçet's disease, injury and alcoholism, intravenous drug abuse, prolonged hospitalization with broad-spectrum antibiotic usage, and HIV were considered predisposing factors for RM in patients. Further, eight of the patients (13%) had a COVID-19 positive history, while six of these patients were treated with corticosteroids. Note that 87% (7/8) of the COVID-19 positive patients died because of the RM. On the other hand, 13 (22%) patients did not have any major risk factors; accordingly, these patients were considered immunocompetent patients. The mortality rate among these patients was 38% (5/13).

Only 22 studies (37%, 22/60) identified species at the species level, where *Rhizopus* spp. was the most frequently isolated pathogen from patients, with nine reports. In this regard, *Rhizopus oryzae* and *Rhizopus microsporus* caused RM in six patients. Further, other Mucorales, including *Apophysomyces elegans* (five cases), *Rhizomucor* spp. (two cases), *Lichtheimia corymbifera* (two cases)*, Absidia corymbifera, Cunninghamella spp, Mycocladus corymbifer*, and *Lichtheimia ramosa* were detected in the patients with RM (Table 1).

**Table 1:** Different clinical aspects of patients with renal mucormycosis (PubMed reported cases 2010 until April 2022).

**Country, year of publication (Reference)**	**Sex/age**	**Pathogen**	**Underling conditions**	**Clinical presentations**	**Antifungals**	**Surgery**	**Diagnosis**	**Outcome**
USA, 2010 1 (18)	61/M	*A. elegans*	Kidney transplantations	Hyperkalemia	LAMB, micafungin	Nephrectomy	HE, molecular and culture	Died
USA, 2010 1 (18)	31/F	*A. elegans*	Kidney transplantations	The decline in urine and fever	LAMB, micafungin	Transplant nephrectomy	HE, molecular and culture	Treated
India, 2010 2 (8)	25/F	NR	Post-partum	Left flank pain, fever and dysuria	NA	NA	HE from autopsy	Died
India, 2010 2 (8)	45/F	NR	Diabetes	Anuria	NA	NA	HE from autopsy	Died
India, 2010 3 (19)	28/M	*NR*	Without any underlying condition	Pain in the left flank and fever	AMB	Splenectomy and left nephrectomy	HE	Treated
Australia. 2010 4 (20)	42/F	*NR*	Alleged assault with injury and alcoholism	Right flank pain	NA	NA	HE from autopsy	Died
Germany, 2010 5 (21)	23/F	*Absidia corymbifera*	Acute lymphocytic leukemia	Right flank pain and fever	LAMB, CASPO and POSO	Right nephrectomy	HE and molecular	Died
India, 2010 6 (22)	17/M	*Apophysomyces elegans*	NR	Flank pain and fever	AMB	Percutaneous nephrostomy	Culture	Treated
India, 2010 6 (22)	32/m	*Mycocladus corymbifer*	NR	Flank pain and fever	AMB	Percutaneous nephrostomy	Culture	Treated
USA, 2010 7 (23)	32/m	*Rhizopus oryzae*	Intravenous drug abuse and diabetes	Oliguria, Flank pain and weight loss	AMB, LAMB and POSO	Cystoureteroscopy with partial extraction	Culture	Treated
India 2010 8 (24)	7/M	*NR*	NR	Flank pain and fever	AMB	Nephrectomy	HE	Treated
Iran, 2012 9 (25)	41/M	*NR*	Liver transplantation	Flank pain and fever	AMB	Nephrectomy	HE and culture	Treated
India, 2012 10 (26)	59/M	*NR*	Diabetic and hypertensive	Leucocyturia and respiratory symptoms	AMB, LAMB	Nephrectomy	HE	Died
China, 2012 11 (27)	59/M	*Rhizopus oryzae*	Kidney transplantation	Light-yellow exudates were seen inside the wound	AMB	Repeated debridement	Culture and molecular	Severe complication
India, 2011 12 (28)	35/M	*Apophysomyces elegans*	NR	Fever, abdominal pain, hematuria.	AMB	Nephrectomy	HE and culture	Died
France, 2013 13 (9)	65/F	*Rhizopus microsporus*	Corticosteroid therapy and tamponade and a percutaneous drainage	Acute renal failure	LAMB, POSO	Debridement of the inguinal abscess	Culture and molecular	Treated
Iran, 2013 14 (1)	1.5/M	*NR*	Neuroblastoma and chemotherapy	Fever and neutropenia	LAMB, POSO	Splenectomy	HE and culture	Died
India, 2013 15 (29)	59/M	*NR*	Kidney transplantation	Fever and dysuria	AMB	Nephrectomy	HE	Treated
Australia, 2013 16 (30)	11/F	*Lichtheimia corymbifera*	T-ALL	Abdominal pain	LAMB, CASPO, POSO	Nephrectomy	HE and culture	Treated
India, 2011 17 (31)	20/M	*NR*	Anemia	Fever, chills and left flank pain	LAMB	NA	HE	Treated
Japan, 2013 18 (32)	14/F	*Rhizomucor*	ALL	Fever and right flank pain	NR	Nephrectomy	HE and culture	Treated
Italy, 2013 19 (33)	26/F	*NR*	Diabetes	Fever and abdominal pain	AMB	Gastrectomy	HE and culture	Treated
India, 2014 20 (34)	1.5/M	*NR*	NR	Fever and pyuria	AMB	Nephrectomy	HE	Treated
USA, 2014 21 (35)	68/F	*NR*	AML on voriconazole prophylaxis	Fever and pneumonia	NA	NA	HE from autopsy	Died
Korea, 2014 22 (36)	54/M	*Rhizopus microsporus*	Diabetes, kidney transplantation	Fever and abdominal pain	AMB	Nephrectomy	HE, molecular and culture	Died allograft kidney infarction
India 2014 23 (37)	34/M	*NR*	NR	Fever and abdominal pain	AMB	Nephrectomy	HE	Died
India, 2015 24 (38)	22/M	*NR*	NR	Fever and pain	AMB	Nephrectomy	HE	Treated
India, 2015 25 (39)	25/M	*NR*	NR	Abdominal pain, fever, hematuria for 3 days and anuria	NA	NA	HE from autopsy	Died
India, 2015 26 (40)	38/M	*NR*	Anemia	High fever, right flank pain, and diarrhea	AMB	Nephrectomy	HE	Treated
USA, 2015 27 (41)	30/M	*NR*	Mediastinal germ cell tumor and chemotherapy	fever and right flank pain	LAMB, POSO	Nephrectomy	HE	Treated
China, 2015 28 (42)	54/M	*NR*	Kidney transplantation	Flank pain and fever	NR	Nephrectomy of the transplanted kidney	HE	Treated allograft kidney infarction
China, 2015 28 b (42)	47/M	*NR*	Kidney transplantation	Flank pain and fever	NR	Nephrectomy of the transplanted kidney	HE	Died allograft kidney infarction
India, 2015 29 (43)	40/M	*NR*	NR	Right flank pain, fever	LAMB	NA	HE	Treated
India, 2016 30 (44)	21/F	*NR*	Anemia	Right flank pain, fever	LAMB	Nasal debridement	HE	Treated
Saudi Arabia, 2017 31 (45)	15/M	*NR*	T-Acute Lymphoblastic Leukemia	Respiratory symptoms	AMB	Nephrectomy	HE	Treated
India, 2017 32 (46)	12/M	*NR*	NR	Fever, right flank pain, and oliguria	AMB	NA	HE	Died
India, 2017 32 (46)	10/M	*NR*	NR	Fever and chills for 15 days, abdominal pain	NA	NA	HE	Died
USA, 2018 33 (47)	57/M	*NR*	Stage-IV sarcoidosis on long-term steroids	GI bleeding and obstructive uropathy	LAMB, ISVA, POSO	NA	HE	Treated
USA, 2018 34 (48)	56/m	*Rhizopus*	Diabetes	Flank pain, fevers	LAMB, POSO	Bladder debris	Culture and HE	Treated
India, 2018 35 (49)	17/F	*Rhizopus*	Idiopathic CD4 lymphocytopenia	Flank pain, fevers	LAMB, POSO	Nephrectomy	Culture and HE	Treated
India, 2018 36 (50)	3/M	*NR*	NR	Flank pain, fevers	LAMB, POSO	NA	HE	Treated
Italy, 2020 37 (51)	15/M	*Lichtheimia corymbifera*	B-NHL and chemotherapy	Fever	LAMB, POSO	Lobectomy	HE and molecular	Treated
Qatar, 2019 38 (52)	35/M	*Apophysomyces elegans*	NR	Left flank pain, hematuria, and fever	LAMB	Nephrectomy	HE and molecular	Died
Netherlands, 2020 39 (53)	38/M	*Lichtheimia ramosa*	Kidney transplantation	Acute kidney failure	LAMB, POSO	Nephrectomy	HE and molecular	Treated nephrectomy of the graft
India, 2021 40 (54)	3 month/M	*NR*	Infant	Right-sided flank lump and fever.	AMB	Tissue debridement	HE	Treated
USA, 2021 41 (55)	3/*	*NR*	Kidney transplantation	Fever, leukocytosis and significant pyuria	AMB, POSO	Nephrectomy	HE	Treated nephrectomy of the graft
India, 2021 42 (56)	32/M	*Rhizopus oryzae*	COVID-19 and steroids therapy	Fever and flank pain	NA	Nephrectomy	HE and culture	Died
South Africa, 2021 43 (57)	39/F	*NR*	HIV	Fever and flank pain	AMB	Nephrectomy	HE	Treated
India, 2021 44 (58)	32/M	*NR*	NR	Fever and flank pain	LAMB	Nephrectomy	HE	Died
India, 2021 45 (59)	32/M	*NR*	COVID-19	Abdominal pain	NA	Nephrectomy	HE	Died
USA, 2021 46 (60)	53/F	*Rhizopus spp*	HCV, HIV and diabetes	Dysuria and hematuria	LAMB, POSO	Nephrectomy	HE	Treated nephrectomy of the graft
India, 2021 47 (61)	32/M	*NR*	COVID-19	Fever and flank pain	NA	Nephrectomy	HE	Died
India, 2022 48 (62)	35/F	*NR*	Kidney transplantation and COVID-19	Fever and oliguria	NA	NA	HE	Died graft
Romania, 2022 49 (63)	7/M	*Cunninghamella spp*	Beta-thalassemia and COVID-19	Fever	NA	Cardiac surgery	Culture and molecular	Died
Japan, 2022 50 (64)	58/M	*Rhizopus microsporus*	COVID-19 and diabetes	Respiratory failure	NA	NA	HE and molecular	Died
India, 2022 51 (65)	32/M	*NR*	COVID-19	Metabolic acidosis and oliguria	AMB and POSO	Nephrectomy	HE and culture	Treated
India, 2022 52 (66)	46/M	*NR*	COVID-19	Fever and left flank pain.	LAMB	Nephrectomy	HE	Died
China, 2022 53 (67)	18/M	*Rhizomucor pusillus*	Philadelphia-like ALL	Various symptoms	LAMB, POSO	NA	Cf-DNA NGS, PCR and HE	Treated
Japan, 2022 54 (7)	76/M	*NR*	Behçet's disease and ileocecal resection	Fever and left flank pain.	NA	NA	HE	Died
India, 2022 55 (68)	54/M	*NR*	Diabetes	Fever and left flank pain.	AMB	Nephrectomy	He and culture	Died

*M, male; F, female; AMB, amphotericin B; LAMB, liposomal amphotericin B; HE, histopathologic examinations; CASPO, caspofungin; POSO, posaconazole; T-ALL, T-cell acute lymphoblastic leukaemias; AML, Acute myelogenous leukemia; ISVA, isavuconazole; GI, Gastrointestinal; B-NHL, B-Cell-Non-Hodgkin lymphoma; HIV, human immune deficiency virus; HCV, hepatitis C virus; Cf-DNA NGS, Cell-free DNA next-generation sequencing; PCR, polymerase chain reaction*.

### Clinical manifestations

Fever (73%, 44/60), flank pain (may be unilateral or bilateral depending upon the extent of disease) (57%, 34/60), oliguria 17% (10/60), hematuria (12%, 7/60), pyuria 10% (6/60), anuria 8% (5/60), and dysuria 5% (3/60) were the most prevalent RM clinical signs reported in the patients. Note that the isolated RM was reported in 57% (34/60) of the cases. Additionally, in five patients, bacterial co-infection was detected: vancomycin-resistant *Enterococcus faecium*, bacterial intraabdominal abscesses, carbapenems resistant *Enterobacter cloacae, Mycobacterium tuberculosis*, and *Pseudomonas aeruginosa*.

### Diagnosis

With respect to laboratory analysis, in 55% (33/60) of the patients, histopathologic examination (HE) alone was sufficient to diagnose RM. Ribbon-like and aseptate hyphae of *Mucor*, with surrounding tissue necrosis, were the most common reported characteristic after HE. Tissue specimens were collected from surgery and biopsy; additionally, in 10 (17%) patients, tissue specimens were collected from an autopsy. Notably, in eight (13%) patients with disseminated mucormycosis, the specimens were collected from other organs apart from kidneys, which included liver, lung, spleen, nasal, gastric, mitral plus tricuspid vegetations, as well as left cervical soft tissue. Furthermore, findings from the combined use of HE + molecular evaluation and HE + culture led to the detection of infection in six (10%) and 12 (20%) patients, respectively. In three (5%) other patients, the combined use of all diagnostic methods (HE + culture + molecular evaluations) resulted in a definite diagnosis of RM.

On the other hand, HE was not used in six (10%) patients. In three of these patients, culture + molecular evaluation led to the diagnosis, while in three other patients, microscopic assessment and culture led to the definite diagnosis. Collectively, positive culture and polymerase chain reaction (PCR) plus sequencing were used to detect infection in 21 (35%) and 11 (18%) patients, respectively. Notably, different specimens, including tissue (15 patients), drainage (three patients), urine (two patients), and pus (one patient), resulted in a positive culture in patients with RM. Nevertheless, blood culture did not identify the *Mucorales* in any of the patients.

Finally, the data about medical imaging modalities, including Computed Tomography (CT) and Ultrasonography, were reported in 67% (40/60) and 47% (28/60) of patients, respectively. The findings of imaging modalities did not yield a conclusive diagnosis for any of the patients. In this regard, in one patient, CT findings led to the suspicion of cancer (38). Nevertheless, among the patients with imaging modalities, enlarged kidney (32%, 19/60), hydronephrosis, and pyelonephritis-like signs (12%, 7/60) were the most common signs reported.

### Treatment

RM has been managed using various approaches. Combined use of surgery and antifungals was the main treatment approach employed in 60% (36/60) of the patients. In addition, surgery alone and antifungal alone were used in 12% (7/60) and 13% (8/60) of the patients, respectively. On the other hand, nine patients (15%) died before any treatment. Notably, among the patients managed without surgery, RM was controlled in three of them with antifungals + drainage without major surgery (22, 43). Another patient was treated with deoxycholate amphotericin B (AMB), but he deteriorated further and he succumbed before surgery (46). In two other patients, surgery was considered by physicians for the management of RM; however, due to the patient's frail state as well as multiple comorbidities and parental opposition, the surgery was not applied to the patients (47, 50).

The most common antifungal agents used in patients with RM were AMB (23 cases, 52%, 23/44), liposomal amphotericin B (LAMB) (22 cases, 50%, 22/44), and posaconazole (17 cases, 39%, 17/44). Note that isavuconazole and echinocandins were used only for two patients (Table 1). The LAMB, posaconazole, and isavuconazole were considered by physicians for the treatment of RM in other patients; however, these antifungals were not available in the clinical setting (26, 57).

Fifty-seven percent (13/23) of patients that had AMB in their treatment regime revealed desired therapeutic results; meanwhile, this drug was ineffective in the rest of the patients, with the LAMB treatment rate being 64% (14/22). Notably, deranged renal function was identified in two patients as AMB adverse events; additionally, renal failure was detected in one patient after LAMB therapy (26, 47, 57).

In two cases, AMB was administered, and both patients recovered completely without surgery (22). On the other hand, in two other patients AMB only caused partial resolution of the mucormycosis mass, and surgical intervention was needed to control the infection (24, 25). LAMB also showed good therapeutic effects in two patients. Both of these patients survived on antifungal therapy alone with additional drainage (31, 43).

Another antifungal agent that was used to treat RM was posaconazole. Sixty-one percent (12/17) of patients treated with this antibiotic showed favorable outcomes. In one patient, the combined used of LAMB and posaconazole (step-down therapy) resolved infection in a 3-year-old boy (50). LAMB (3 mg/kg/day) was used for the treatment of disseminated mucormycosis in another patient with ALL. This antifungal inhibited the infection, but CT indicated no significant decrease in the focus of the liver, spleen, and kidneys, but a new lesion appeared in his brain. Posaconazole combined with LAMB and CT (at the 4 and 8th week of the combination antifungal treatment) indicated that all lesions in the mentioned organs significantly decreased. Collectively, the patient had a desirable outcome through treatment with LAMB sequential posaconazole (67).

Nevertheless, the use of posaconazole for the treatment of three patients with RM failed due to some issues. For example, in one patient, the physicians were not able to demonstrate acceptable systemic posaconazole blood concentrations despite aggressive attempts to maximize absorption (18). In another patient, posaconazole was discontinued after 3 months because of the failure to achieve satisfactory therapeutic levels (30, 31). Finally, this antifungal was avoided initially because the patient was on cyclosporine, while posaconazole is a CYP enzyme inhibitor, which could lead to the drug interaction with cyclosporine (31).

Isavuconazole, one of the triazole class antifungal agents, was used to treat RM in only two patients. Both of these patients recovered from the infection. In one of these patients, a surgical approach was considered; however, given the patient's multiple comorbidities and frail state, such an approach was deferred. Additionally, LAMB was switched to oral posaconazole due to the deterioration of renal function. Nevertheless, posaconazole did not control the infection well; accordingly, treatment was switched to intravenous isavuconazole at a dose of 372 mg every 8 h. The patient completed 6 months of isavuconazole therapy, whose kidney function improved and remained stable (47).

Surgery was another approach that was used for the treatment of patients with RM. Different types of surgery were performed on 72% (43/60) of patients, and in 61% of them, a surgical procedure was necessary to inhibit the infection. Nephrectomy was the most surgical intervention that was used on the patient. In one case, AMB was discontinued due to renal failure, and nephrectomy managed the infection (57). In two other patients, treatment with AMB only led to the partial resolution of the mass, and nephrectomy controlled the infection (24, 25).

**Figure 2:**
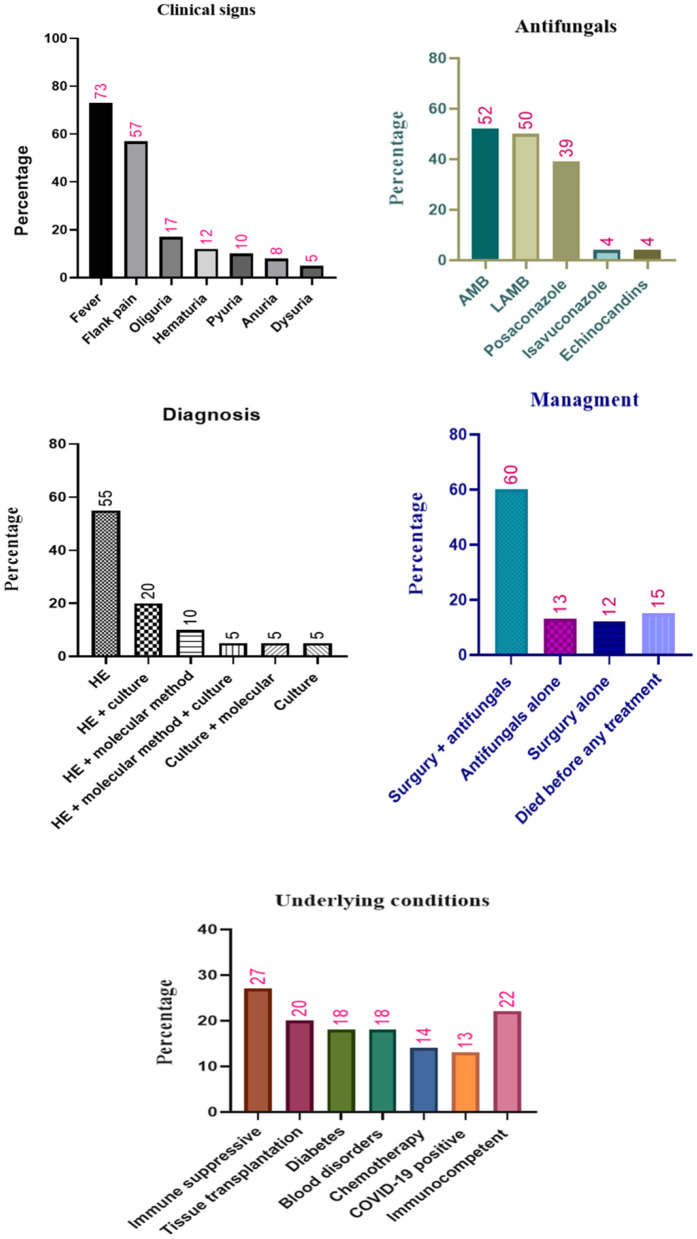
Different characteristics and antifungals treatment of patients with renal mucormycosis. HE, histopathologic examination; AMB, amphotericin B; LAMB, liposomal amphotericin B.

For better understanding, all mentioned findings are presented in Figure 2.

## Discussion

Kidney involvement is one of the rare forms of mucormycosis with a high rate of mortality. Our analysis showed a 44% mortality rate in patients with RM (PubMed reported cases 2010–2022). Our recent investigations also showed 46.3 and 44% mortality rates in patients with ROCM and GI mucormycosis (2, 15). As with GI mucormycosis and ROCM, India had the highest incidence of RM. The high prevalence of diabetes in India is one of the most important reasons for such a high prevalence of mucormycosis in this country. Additionally, some other factors such as socio-economic situation, scarce hygienic conditions, climate, malnutrition, and lack of early diagnosis of mucormycosis can elevate this infection mortality rate in Indian patients. Nevertheless, deficiencies of the health care facilities have caused a broad range of the population of Indian diabetic patients to remain undiagnosed and uncontrolled; it seems that screening and controlling diabetes could be a useful tool to control the spread of mucormycosis in this country (69–71). Furthermore, the lack of effective drugs against mucormycosis such as LAMB, Posaconazole, and isavuconazole in low-income countries could increase the mortality rate of mucormycosis (26). Thus, better management of diabetes and the use of the most effective antifungal against mucormycosis could control this infection in patients from low-income countries.

Our investigation indicated that tissue transplantations, especially kidney transplantation, are among the most common related risk factors in patients with RM. The occurrence of mucormycosis in renal transplant is associated with various factors such as increased usage of immunosuppression in the initial post-transplant period with induction protocols and antirejection therapy such as anti-lymphocyte antibodies, pulse steroids, and interleukin (IL)-2 receptor antagonists, as well as intraoperative or postoperative surgical complications. Further, infected organ transmissions and the existence of immunomodulating viruses such as hepatitis C and cytomegalovirus should also be considered (29, 72). In this regard, a recently published study suggested that metabolic disorders and the nutritional status of the patients requiring tissue transplantation should be improved. Recipients and donors should also be strictly selected to reduce the risk of graft rejection, and antifungal drugs could be added to the renal perfusion fluid before surgery. Additionally, operation room condition is also very important since it could lead to tissue contamination during kidney collection, kidney perfusion, and transplant operation (42).

As with transparent patients, the use of corticosteroids in COVID-19-positive patients also increased the prevalence of mucormycosis in three recent years. The results of the present study also showed an 87% mortality rate for RM in COVID-19-positive patients. Coronavirus causes ciliary dysfunction, cytokine storm, microvascular hypercoagulability, and consequently innate immune dysregulation. Anti-IL-6-directed therapies and corticosteroid treatment that are used for the treatment of COVID-19 patients lead to immune suppression. Additionally, other risk factors such as mechanical ventilation, invasive procedures, industrial-grade oxygen administration, as well as prolonged hospital stays provide the perfect setting for invasive mucormycosis (56, 64, 73). Collectively, during COVID-19, systemic steroid treatments and uncontrolled diabetes are the main risk factors for RM. Thus, physicians should always be cautious of this infection in patients suffering from severe COVID-19 who are treated with extensive immunosuppressive therapy (64). Notably, *Mucorales* have an ability for blood invasion; thus, these fungi are associated with infarction and embolism. However, thrombosis is diagnosed frequently in COVID-19 patients in the critical care unit. Thus, it remains difficult to clinically distinguish SARS-CoV-2-associated thrombosis from mucormycosis-associated thrombosis where the possible presence of mucormycosis should be considered by the physicians in the patients presenting with sequential multiple thromboses (59, 64).

The results of the present investigation indicated that 22% of the patients with RM did not have any mucormycosis-associated risk factors. One study reported that the use of multiple antibiotics prior to admission for the treatment of urinary infection could be related to the development of RM (35). Additionally, hospital-acquired mucormycosis should be considered because of the contaminated air filters and wound dressings, administration of intravenous fluid contaminated with the fungus, and heavy air fungal loads of construction, as well as even tongue depressors (39, 44). Thus, RM also should be considered in immunocompetent patients who have been hospitalized for a long time.

Fever, flank pain, and oliguria were reported as the most commonly RM symptoms in the literature. Thus, this infection, in most cases, shows non-specific clinical manifestations which are comparable to those of other diseases such as other fungal infections, tuberculosis, and renal cell carcinoma (38). However, timely diagnosis is essential for early therapeutic intervention to limit dissemination and progressive angioinvasion, reduce the extent of surgical resection, as well as improve survival and outcome (30).

Imaging modalities have a contributory role in detecting the RM, but the diagnosis cannot be established merely on imaging. Nevertheless, perinephric stranding and enlarged non-hydronephrotic kidneys with hypodensities, as well as areas of low attenuation and perinephric fluid collection, are common findings in contrast-enhanced CT in patients with RM. Additionally, the combined use of Doppler ultrasonography and Magnetic resonance imaging (MRI)/CT could enhance the efficacy of RM diagnosis (1, 28, 39). Thus, physicians should be aware and fungal etiology must be considered after observing the mentioned signs.

In most cases, HE, the gold standard for ruling out and confirming the mucormycosis diagnosis, leads to the definitive diagnosis of RM. *Mucorales* are recognized by their broad aseptate hyphae branching at right angles at irregular intervals. Slender, dichotomously branching and septate could indicate *Fusarium, Scedosporium*, or *Aspergillus* species. Yeast with pseudohyphae may be associated with infections caused by *Candida* species (74–76).

However, unlike other pathogenic fungi which strongly react with histochemical stains, *Mucorales* are often difficult to detect. In this regard, pathologists should maintain a high index of suspicion for invasive mucormycosis, particularly in recipients with clinical symptoms compatible with an infectious process (18, 19). As mentioned, RM must be differentiated from cancer, tuberculosis, and other fungal infections. Atypical malignant cells could differentiate RM from renal cell carcinoma in HE. Nevertheless, fungal infections and tuberculosis both have multinucleated giant cells, neutrophil and macrophage infiltration, as well as granulomas. In this regard, the exact diagnosis of tuberculosis and fungal infections could be achieved from positive acid-fast bacilli and the presence of fungal hyphae in periodic acid-Schiff stain or Gomori silver methenamine, respectively (38).

Furthermore, our evaluations indicated that culture helped detect RM in 30% of cases and the chance of isolating *Mucorales* when biopsies or surgery specimens were used was higher for this method. Notably, culture could not differentiate between invasive infection and harmless contamination without repeated isolation of the fungus from sterile body sites (77). Further, several days were needed to identify the fungus species even after detecting a mold on the culture media (78). Accordingly, HE remains the gold standard for the detection of invasive mucormycosis.

However, in different patients, such as hematological patients, the use of surgery or biopsy to collect tissue specimens is almost unfeasible, and due to severe conditions, empirical antifungal therapy is started before biopsy, which would lower the chance of distinguishing the different fungal pathogens (67, 79).

To this end, the use of molecular methods has been considered for the diagnosis of RM in recent years. The present study showed that only 11 cases were studied using molecular methods based on PCR amplification and sequencing of the large nuclear subunit (28S) along with small nuclear subunit (18S) rRNA genes, as well as the internal transcribed spacer (ITS) region of rRNA. Molecular methods could facilitate an early diagnosis of RM and detect infrequently isolated organisms. Further, quantitative PCR (qPCR) could be used within 7 days after the initial treatment to monitor the response to antifungals. A negative qPCR indicates the response to the treatment (80, 81). However, molecular methods have a high cost, are not used in many laboratories, the positive threshold is not strictly defined and still, large-scale data are required in order to verify their specificity (27, 67). Collectively, combining molecular methods and HE can lead to an accurate and timely diagnosis of RM where culture is needed to ascertain microbial susceptibility to various antifungal agents.

After a timely and accurate diagnosis, the use of appropriate antifungals is essential in the treatment of RM. AMB and LAMB, based on the availability of antifungal drugs, are suggested for mucormycosis treatment as a first-line treatment regime (82). Our assessment also indicated that the mentioned antifungals had been widely used in patients with RM. LAMB showed better efficiency and fewer adverse event in comparison to the AMB. However, the use of LAMB is mainly contingent on the availability of antifungal agents in the clinical setting (83).

Furthermore, delayed-release tablets or intravenous posaconazole and intravenous isavuconazole are recommended for the treatment of mucormycosis (82). Our results indicated acceptable efficacy for posaconazole in the treatment of RM. However, this antifungal was used in only two patients, and further studies are required for better evaluation of isavuconazole efficacy in RM treatment. Posaconazole was also used in 17 patients and demonstrated good performance in managing Polymerase chain reaction (PCR) in some patients (23, 50, 67). However, this antifungal, as with LAMB and isavuconazole, is not available in low-income countries (26). On the other hand, these antifungals did not show an acceptable therapeutic effect in five patients (1, 18, 21, 30, 47). In this regard, the concentration of posaconazole should be monitored in patients at the beginning of the treatment, where a low concentration of this antifungal could lead to initial therapeutic failure. The resultant bioavailability and absorption of posaconazole are best achieved when taken with a high-fat meal (9, 30).

In 72% of patients, surgery was another therapeutic approach. *Mucorales* have an angioinvasive ability; thus, mucormycosis leads to the thrombosis of small and large arteries and tissue necrosis. In this regard, mucormycosis would reduce the penetration of antifungals and leukocytes to the site of interest; accordingly, the removal of infected and devitalized tissue by surgical intervention could be effective (84).

Finally, it should be noted that some patients died despite the appropriate use of surgery and antifungals (1, 21). Thus, management of a patient's underlying condition is essential in the treatment of RM. For instance, a patient died due to the RM despite intensive treatment (surgery + AMB and posaconazole). This patient had a history of allogeneic stem cell transplantation and suffered from graft-vs. -host disease; accordingly, if immune reconstitution cannot be achieved, successful eradication of invasive mucormycosis is rarely possible (21). Immunosuppressant impair the interferon gamma-driven response to fungal pathogens, decrease neutrophils and macrophage phagocytic function, as well as impair their migration. Hence, if it is possible, the immunosuppressive regimen should be withdrawn.

### Limitations

The present study included only PubMed/MEDLINE studies available in the English language, and that contained an abstract, which may have reduced the number of relevant publications. Only individual case reports were included in the present review, and the observational studies were excluded because of their insufficient or absence of information. In addition, many studies have not reported information on the type of microorganism that causes the infection. Details of treatment such as reasons for choosing the drug, dose, duration, adverse events, and drug interactions for treatment of this subset of patients were not reported in some studies. It's noteworthy to mention that case reports and case series are more likely to be biased than other studies; additionally, these studies are descriptive and describe the patient's signs and symptoms. The prevalence and percentage of co-infection in them have not been studied. Finally, it was not possible to discuss the bias, risks, or individual limitations of the studies since these were not reported.

## Conclusion

Renal involvement is a rare form of invasive mucormycosis with a high mortality rate. Our results showed that *Rhizopus* spp. was the most frequently isolated pathogen from patients. COVID-19 has increased the prevalence of mucormycosis in recent years, and management of corticosteroid therapy is critical in COVID-19-positive patients. The early diagnosis of RM is necessary; accordingly, the combined use of imaging, HE, and molecular methods could enhance the efficacy of diagnosis. In addition to timely diagnosis, withdrawal of immunosuppressant, appropriate surgical intervention and antifungal therapy plus immunotherapy with interferon-gamma, as well as bacterial superinfection management are the main factors related to the successful outcome in RM. Noteworthy, some antifungal such as LAMB, isavuconazole, and posaconazole are mostly used by rich countries, and perhaps providing conditions for wider use of these treatments can be effective in controlling RM in scarce hygienic conditions. Additionally, a multidisciplinary team consisting of an expert physician, radiologist, histopathologist, surgeons, and microbiologist is frequently needed for the successful management of RM.

## Data availability statement

The authors confirm that the data supporting the findings of this study is available within the article and its supplementary material.

## Author contributions

AS and MD conceived and designed the study, as well as analyzed the cases. AS, AK, and AM contributed to comprehensive research. AS, MD, and ZC wrote the paper. Notably, all authors have read and approved the manuscript.

## Conflict of interest

The authors declare that the research was conducted in the absence of any commercial or financial relationships that could be construed as a potential conflict of interest.

## Publisher's note

All claims expressed in this article are solely those of the authors and do not necessarily represent those of their affiliated organizations, or those of the publisher, the editors and the reviewers. Any product that may be evaluated in this article, or claim that may be made by its manufacturer, is not guaranteed or endorsed by the publisher.
